# Oral Health and Dietary Habits Before and After COVID-19 Restrictions in a Portuguese Adult Population: An Observational Study

**DOI:** 10.3390/life15050746

**Published:** 2025-05-06

**Authors:** Eduardo Guerreiro, Ricardo Cachinho, Tiago Dionísio, Manuel Nobre, André Júdice, Cátia Simões, José João Mendes

**Affiliations:** 1 Egas Moniz Center for Interdisciplinary Research (CiiEM), Egas Moniz School of Health & Science, 2829-511 Almada, Portugal; rcachinho@egasmoniz.edu.pt (R.C.); tdionisio@egasmoniz.edu.pt (T.D.); mnobre@egasmoniz.edu.pt (M.N.); ajudice@egasmoniz.edu.pt (A.J.); csimoes@egasmoniz.edu.pt (C.S.); jmendes@egasmoniz.edu.pt (J.J.M.); 2 iBB-Institute for Bioengineering and Biosciences, Department of Bioengineering, Instituto Superior Técnico, Universidade de Lisboa, Av. Rovisco Pais, 1049-001 Lisboa, Portugal; 3 Associate Laboratory i4HB–Institute for Health and Bioeconomy, Instituto Superior Técnico, Universidade de Lisboa, Av. Rovisco Pais, 1049-001 Lisboa, Portugal

**Keywords:** COVID-19, oral health, oral health habits, dietary habits, pandemic, COVID restrictions

## Abstract

**Background:** The declaration of COVID-19 as a pandemic by the World Health Organization in 2020 led to the widespread suspension of clinical practices, including dentistry. This study aims to evaluate the impact of these restrictions on oral health and dietary habits. **Methods:** A retrospective cross-sectional study was conducted at Egas Moniz University Clinic (Lisbon Metropolitan Area), covering from June 2019 to June 2021. A total of 3380 participants were included and categorized into two cohorts: pre- and post-COVID-19 restrictions. Data were collected through a structured questionnaire assessing oral health behaviors and dietary habits. **Results:** Of 3469 incoming patients, 3380 met the inclusion criteria. Statistically significant post-lockdown changes were observed, including increased smoking prevalence, higher coffee with sugar consumption, reduced use of dental floss and mouthwash, and redistribution in tooth brushing frequency, with fewer individuals brushing 2–3 times daily. **Conclusions:** COVID-19-related restrictions had a heterogeneous impact on oral health and dietary behaviors. While some individuals reported improved hygiene routines, others showed negative changes, such as increased tobacco use or decreased use of oral hygiene products. These contrasting effects call for targeted public health strategies to reduce inequalities and support vulnerable groups during crises.

## 1. Introduction

In 2020, the World Health Organization (WHO) declared COVID-19 a pandemic [1]. The uncertainty regarding the transmission of the coronavirus and concerns about its spread led health authorities worldwide to suspend certain clinical practices, including dentistry [2].

In Portugal, the government declared a national state of alert on 13 March 2020, which quickly escalated into a nationwide lockdown. On 16 March 2020, the Ministry of Health issued Ministerial Order no. 3301-A/2020, suspending all non-urgent dental, stomatological, and odontological activities, allowing only emergency care. This measure drastically limited access to routine oral healthcare across the country, including in the Lisbon Metropolitan Area, where this study was conducted. Dental clinics remained closed until 18 May 2020, when Ministerial Order no. 3903-E/2020 authorized the gradual reopening of dental services under strict health and safety protocols.

Consequently, the population experienced a two-month interruption in routine dental care, followed by a gradual adaptation to new clinical guidelines. This disruption, combined with broader lockdown measures, contributed to significant changes in daily routines and eating habits. Increased reliance on remote work, a rise in home-delivered fast-food consumption, and postponed dental visits raised the risk of oral health problems.

This pandemic response fundamentally altered the way dental care was delivered. Many dental offices and healthcare institutions, adapting to social isolation measures, implemented new treatment protocols and were limited to providing only urgent and emergency care [3,4].

Globally, the COVID-19 pandemic disrupted everyday habits, including diet and oral hygiene. People working remotely often turned to unhealthy foods and snacks, and delayed their regular dental appointments, further increasing the risk of oral health issues [5]. As a result, patient care underwent significant changes, with unclear consequences for oral hygiene and general oral health [6,7].

In this context, dental clinics have played a key role in promoting and reinforcing oral hygiene practices. Regular check-ups allow professionals to educate patients on proper brushing and flossing techniques, which are often neglected in daily routines, and help mitigate the negative impacts of restricted access to care [7].

The lockdown periods also had a marked effect on mental health and emotional well-being [5]. Under stress, individuals often adopt unhealthy coping behaviors [8,9]. Long periods spent at home, often marked by boredom and anxiety, led many to increase tobacco use [10,11] and consume more meals, snacks, and comfort foods, typically high in sugar [12,13,14,15]. On the other hand, the additional time spent at home could have promoted greater care with oral hygiene routines, although this was not consistently observed.

A noticeable shift in dietary habits emerged, with increased consumption of sugary foods and a general decline in oral hygiene behaviors. These lifestyle changes may contribute to a higher incidence of oral diseases, including dental caries [16].

Despite this context, the impact of COVID-19-related restrictions on oral health behaviors remains insufficiently explored. To address this gap, we conducted a retrospective analysis of first-time patients attending a reference dental clinic at a Portuguese university, aiming to understand the effects of pandemic-related restrictions on their oral health and dietary habits. The null hypothesis (H0) tested in this study is that the restrictions imposed during the COVID-19 pandemic had no significant impact on oral health or dietary behaviors.

## 2. Materials and Methods

This study was conducted in accordance with the STROBE guidelines for the reporting of observational studies in epidemiology [17], and with the ethical principles set out in the Declaration of Helsinki (1975), as last revised in 2024. Ethical approval was obtained from the Institutional Review Board of Egas Moniz (ID no. 898, issued on 24 September 2020). All participants provided written informed consent at the time of their initial appointment.

### 2.1. Study Setting and Participants

This study was designed as a retrospective analysis of responses obtained from a questionnaire completed by new patients during their first consultation, using a comprehensive database of patients who attended the clinic between June 2019 and June 2021.

The questionnaire used in this study was the standardized instrument recommended by the World Health Organization in the publication Oral Health Surveys: Basic Methods (5th Edition), composed of 16 questions [18]. This internationally validated tool includes a structured set of closed-ended questions that assess sociodemographic characteristics, medical history, tobacco use, oral hygiene behaviors, and dietary habits.

The initial section of the questionnaire collected demographic data such as sex, age, employment status, educational level, medical history, smoking habits, and oral hygiene practices.

Inclusion criteria required participants to be aged 18 years or older and to provide informed written consent. Total edentulism was an exclusion criterion.

Participants were divided into two groups based on the date of questionnaire completion: Group 1 (BCR)—individuals assessed before the implementation of COVID-19 restrictions (from June 2019 to early March 2020), and Group 2 (ACR)—individuals assessed after the easing of restrictions (from May 2020 to June 2021).

### 2.2. Dependent Variables

Oral hygiene habits were assessed based on the frequency of tooth brushing (2–3 times daily, once per day, 2–6 times per week, or never), the choice between a manual and an electric toothbrush, as well as the use of dental floss and mouthwash.

Additionally, dietary habits were examined, including the consumption of fresh fruit, biscuits, cakes, sweets or candies, lemonade or other soft drinks, coffee with sugar, and tea with sugar. The frequency of consumption was categorized as several times a day, daily, several times a week, once a week, several times a month, or rarely/never.

### 2.3. Independent Variables

Sociodemographic data were collected through a self-administered questionnaire completed before the clinical assessment to minimize potential response bias.

The independent variables included key indicators related to health determinants and sociodemographic characteristics, specifically gender, age, educational attainment, body mass index (BMI), and smoking habits. Gender was categorized as male or female. Age was initially recorded as a continuous variable (in years) and subsequently grouped into four categories: 18–24, 25–44, 45–64, and 65 years or older. Educational attainment was classified according to the 2011 update of the International Standard Classification of Education (ISCED). This classification methodology aligns with previous studies evaluating similar variables [19,20,21].

### 2.4. Statistical Analysis

Data analysis was conducted using IBM SPSS Statistics version 28.0 (IBM Corp., Armonk, NY, USA). Descriptive statistics were computed to summarize demographic, clinical, and behavioral data. The normality of continuous variables was assessed using the Kolmogorov–Smirnov test. Continuous variables were expressed as mean ± standard deviation (SD) or median (interquartile range), depending on the distribution.

Inferential analysis was performed using independent samples *t*-tests to evaluate differences between the two groups (before and after COVID-19 restrictions) regarding continuous and ordinal variables. Categorical variables were compared using chi-square tests. For the purposes of statistical testing, categorical and ordinal variables were transformed into numerical codes: binary variables (e.g., smoking status, dental floss usage, mouthwash usage) were coded as 0 (No) and 1 (Yes), while ordinal variables (e.g., tooth brushing frequency, dietary habits) were coded using ascending integer values based on frequency (e.g., 1 = never, 2 = several times a month, …, 6 = several times a day). This numeric transformation allowed for the comparison of group means using *t*-tests.

Additionally, Spearman’s rank correlation coefficient was used to explore potential associations between sociodemographic factors (age, gender, education) and behavioral variables (e.g., smoking status, BMI, oral hygiene, and dietary habits), given the ordinal nature and potential non-normal distribution of the data.

Statistical significance was set at *p* < 0.05.

## 3. Results

### Participants’ Inclusion and Characteristics

**Figure 1:**
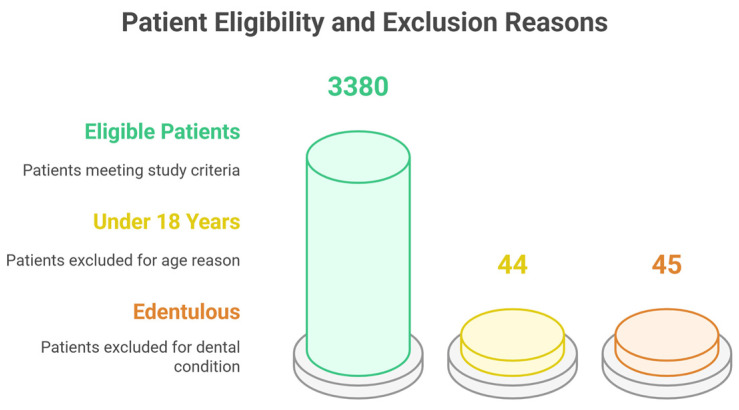
Participants inclusion.

Of the 3469 patients initially assessed, 3380 (97.4%) met the eligibility criteria and were included in this study. A total of 89 individuals were excluded: 44 (1.3%) were under 18 years of age, and 45 (1.3%) were edentulous (Figure 1).

Table 1 presents a detailed sociodemographic, health, and behavioral profile of the 3380 participants, divided into two groups: pre-lockdown (*n* = 2278) and post-lockdown (*n* = 1102). Overall, females represented 59.4% of participants, decreasing slightly from 60.1% pre-lockdown to 56.8% post-lockdown. Conversely, males accounted for 40.6%, showing an increase from 39.9% pre-lockdown to 43.2% post-lockdown.

The mean age of participants was 43.21 years, with a minor decline from 43.36 years pre-lockdown to 42.90 years post-lockdown. Age distribution revealed that 21.3% were in the 18–24 years category, 32.4% were aged 25–44 years, 32.2% belonged to the 45–64 years group, and 14.2% were 65 years or older.

Regarding education levels, participants without formal education comprised 22.4%, showing a reduction from 24.1% pre-lockdown to 18.7% post-lockdown. Elementary education was reported by 37.4%, slightly decreasing from 37.7% pre-lockdown to 36.8% post-lockdown. Middle-level education was indicated by 26.9% of participants overall. Higher education notably increased from 0.7% pre-lockdown to 8.7% post-lockdown, with an overall percentage of 3.3%.

In terms of Body Mass Index (BMI), 3.6% of participants had a BMI below 18.5 kg/m^2^. The majority (46.1%) had a BMI within the normal range (18.5–24.9 kg/m^2^), increasing from 44.5% pre-lockdown to 49.5% post-lockdown. Those classified as overweight (25.0–29.9 kg/m^2^) represented 34.1%, with minimal variation between periods. Participants classified with obesity (BMI ≥ 30.0 kg/m^2^) accounted for 16.2% overall, decreasing from 17.3% pre-lockdown to 14.0% post-lockdown.

Lastly, 79.2% of the participants reported being non-smokers. However, there was a notable increase in active smokers from 18.0% pre-lockdown to 26.6% post-lockdown.

**Figure 2:**
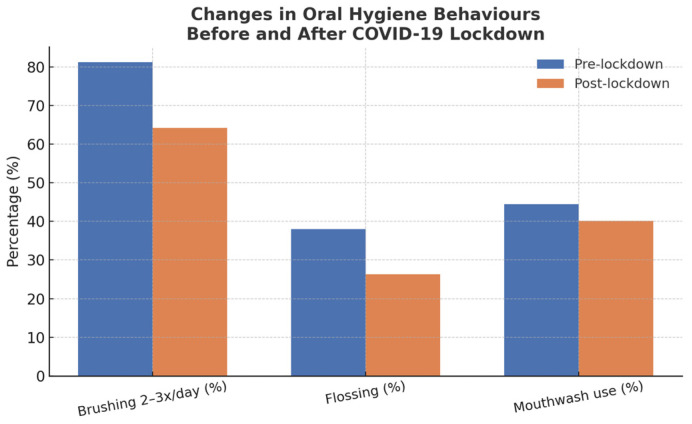
Comparative bar chart of brushing frequency, flossing, and mouthwash use before and after the COVID-19 lockdown.

Table 2 summarizes oral hygiene practices of the 3380 participants, divided into pre-lockdown (*n* = 2278) and post-lockdown (*n* = 1102) groups. Figure 2 presents a bar chart illustrating the differences in oral hygiene behaviors before and after the COVID-19 lockdown, highlighting changes in brushing frequency, flossing, and mouthwash use.

The majority (88.6%) of participants used manual toothbrushes, with minimal variation between periods (88.5% pre-lockdown vs. 88.6% post-lockdown). Electric toothbrush usage was relatively low, representing 10.9% overall, with a slight increase from 10.8% pre-lockdown to 11.1% post-lockdown.

Regarding tooth brushing frequency, 75.6% brushed their teeth 2–3 times daily overall, although this percentage significantly decreased from 81.2% pre-lockdown to 64.2% post-lockdown. Participants brushing once daily accounted for 15.0%, slightly declining from 15.5% pre-lockdown to 14.1% post-lockdown. Notably, those brushing 2–6 times weekly increased substantially from 1.9% pre-lockdown to 18.5% post-lockdown, with an overall average of 7.3%. Participants who never brushed their teeth constituted 2.1%, rising from 1.5% pre-lockdown to 3.2% post-lockdown.

Dental floss was not commonly used, with 65.8% of participants reporting no flossing, increasing from 62.0% pre-lockdown to 73.7% post-lockdown. Correspondingly, dental floss use decreased from 38.0% pre-lockdown to 26.3% post-lockdown, with an overall usage of 34.2%.

Mouthwash use was reported by 43.1% of participants overall, with a decrease from 44.5% pre-lockdown to 40.1% post-lockdown. Conversely, those not using mouthwash increased slightly from 55.5% pre-lockdown to 59.9% post-lockdown, totaling 56.9% overall.

Table 3 provides descriptive data regarding the dietary habits of the 3380 participants.

Fresh fruit consumption was frequent among participants, with 44.7% reporting consumption several times a day, increasing slightly from 43.8% pre-lockdown to 46.7% post-lockdown. Daily fruit consumption remained stable at around 30.5%. A small percentage (3.8%) seldom or never consumed fresh fruit.

Regarding biscuits and cakes, 44.1% of participants reported weekly consumption, with little variation between periods. Daily consumption decreased slightly from 18.8% pre-lockdown to 15.6% post-lockdown. Conversely, those seldom or never consuming biscuits and cakes increased from 26.9% pre-lockdown to 29.7% post-lockdown.

Daily honey consumption showed a small increase from 7.0% pre-lockdown to 9.1% post-lockdown, and once a week consumption increased from 19.4% pre-lockdown to 21.1% post-lockdown.

Sweets or candies had weekly consumption reported by 44.1% of participants overall, increasing slightly post-lockdown (45.6%) compared to pre-lockdown (43.4%). Daily intake decreased from 13.2% pre-lockdown to 12.3% post-lockdown, while occasional consumption (several times a month) slightly increased post-lockdown.

The majority (54.6%) of participants reported seldom or never consuming lemonade or soft drinks, with little variation between pre-lockdown (54.3%) and post-lockdown (55.1%). Daily consumption decreased from 10.2% pre-lockdown to 8.5% post-lockdown.

A total of 78.1% of participants rarely or never added sugar to their tea. Regular tea consumption (daily or several times daily) was stable at around 9.8% overall, with minimal variation between the two periods.

Coffee with sugar was consumed several times a day by 21.0% of participants overall, decreasing from 22.5% pre-lockdown to 18.1% post-lockdown. Notably, the percentage of participants who seldom or never drank coffee with sugar decreased from 54.0% pre-lockdown to 47.7% post-lockdown. Additionally, there was a substantial increase in missing or non-responses post-lockdown (16.2%).

Table 4 presents the results of independent samples *t*-tests comparing oral health and dietary habits between the pre-lockdown (*n* = 2278) and post-lockdown (*n* = 1102) groups. To enable statistical comparison, categorical and ordinal variables were transformed into numerical scores, as described in the Statistical Analysis section. For instance, binary variables such as smoking status or dental floss usage were coded as 0 (No) and 1 (Yes), while ordinal variables such as dietary frequency or tooth brushing were assigned ascending values based on frequency.

Table 4 presents the results of independent samples *t*-tests comparing oral health and dietary habits between the pre-lockdown (*n* = 2278) and post-lockdown (*n* = 1102) groups. The transformation of categorical and ordinal variables into numerical scores for statistical analysis is detailed in the Statistical Analysis section.

To further explore the potential influence of sociodemographic characteristics on health-related behaviors during the pandemic, a correlation analysis was conducted using Spearman’s rank correlation coefficient. This non-parametric method was selected due to the ordinal nature of several variables and the absence of normal distribution in some cases. The analysis focused on age, gender, and education level in relation to selected behavioral variables, including smoking status, body mass index (BMI), and dietary and oral hygiene habits.

Table 5 presents the statistically significant correlations observed. Notable findings include a negative association between education and BMI, a positive correlation between education and smoking status, and a negative correlation between age and honey consumption, among others.

## 4. Discussion

This retrospective study assessed oral health habits and behaviors in a Portuguese adult population using self-reported questionnaires completed during initial dental consultations. The main goal was to evaluate how oral health habits were affected by the COVID-19-induced lockdown by comparing self-reported practices and attitudes before and after this period across various demographic and behavioral factors.

Our results revealed statistically significant changes in several behaviors following the lockdown, including an increase in smoking prevalence, a decrease in flossing and mouthwash use, and a shift in toothbrushing frequency, with fewer individuals brushing their teeth 2–3 times per day. During the COVID-19 pandemic, restrictive policies such as lockdowns, social distancing, and isolation significantly impacted daily routines, mental health, economic stability, and lifestyle habits, potentially influencing smoking behaviors and patterns among individuals [22].

The implementation of lockdown measures significantly impacted lifestyle behaviors, including smoking habits. Evidence from recent studies indicates that social isolation and psychological stress associated with home confinement led to increased tobacco consumption [22,23,24]. In Italy, a web-based cross-sectional study conducted during the lockdown revealed that approximately 30% of smokers increased their cigarette consumption by an average of 5.6 cigarettes per day, while a small proportion (0.6%) of former smokers resumed smoking [23]. This shift towards increased smoking aligns with a systematic review and meta-analysis showing that, despite varied individual responses, a considerable number of smokers globally reported heightened tobacco use during the pandemic period [24].

Furthermore, the COVID-19 lockdown conditions not only increased smoking frequency among existing smokers but also influenced smoking initiation, particularly among vulnerable groups. A cross-sectional study in Jordan documented that 22.4% of smokers started smoking during the year of the pandemic lockdown, with young adults and females significantly more likely to begin smoking in response to the stress factors related to COVID-19 restrictions [22]. Similarly, in Portugal, individuals experiencing sustained high levels of stress, anxiety, and depression during lockdown were more likely to be smokers, suggesting tobacco use as a coping mechanism during pandemic-related restrictions [25]. The pandemic thus exacerbated unhealthy lifestyle choices, highlighting the critical need for targeted public health interventions to address increased tobacco use resulting from pandemic-related stress and social isolation.

Despite the observed mean reduction in honey consumption from the pre-lockdown to the post-lockdown group in our data, recent literature reports contrasting findings. Studies suggest that the COVID-19 pandemic has led to an increased interest in honey consumption, particularly organic honey, due to heightened consumer awareness regarding health, food safety, and sustainability [26,27]. Consumers viewed honey as a genuine, natural, and health-promoting product, integrating it more frequently into their diets to support immune function and overall well-being during and following the pandemic period. Thus, while our observations highlight a decline, existing research underscores continued and potentially enhanced appeal of honey as a beneficial food in the context of COVID-19 [28].

Our findings indicate a decrease in coffee consumption, particularly coffee with sugar, after the COVID-19 pandemic restrictions. However, other studies have shown different trends. Some research suggests that coffee consumption increased during the pandemic, possibly due to changes in daily routines and higher stress levels from social isolation [29,30,31]. In Portugal, the COVID-19 lockdown led 8.7% of people to report an increased need for stimulant drinks, primarily coffee, suggesting its role as a coping strategy in stressful times [32]. In contrast, other authors found that although spending on coffee declined, the daily amount consumed slightly increased [31]. These contrasting findings highlight the complexity of consumption behavior shifts caused by the pandemic, underscoring the importance of considering broader psychosocial and economic factors when interpreting changes in dietary habits.

Regarding oral hygiene practices, dental floss usage significantly declined by 12% (*p* < 0.001), while mouthwash usage also decreased slightly yet significantly (*p* = 0.015). Tooth brushing frequency significantly improved (from 0.24 to 0.62, *p* < 0.001).

It is important to note, however, that this result is based on the numerical transformation of an ordinal scale used for statistical testing. Although the mean value on this scale increased, the proportion of individuals who reported brushing their teeth 2–3 times daily actually decreased from 81.2% pre-lockdown to 64.2% post-lockdown, as shown in Table 2. This suggests redistribution across categories rather than a straightforward improvement. These findings suggest varied impacts of the COVID-19 lockdown on dietary habits and oral health behaviors, reinforcing previous research highlighting the influence of the pandemic on lifestyle choices [6,33]. Similar trends were observed in other studies [34], which reported increased self-reported oral health care issues such as dental plaque accumulation and tooth discoloration due to poor oral hygiene and altered dietary patterns during the lockdown period. Other studies further supported these findings, noting that dietary habits, particularly increased consumption of sweets and snacks, negatively affected oral health during confinement [35]. Additionally, a systematic review emphasized that reduced access to routine dental care and increased stress led to decreased oral hygiene practices, further exacerbating oral health problems during the COVID-19 pandemic [36].

The correlation analysis further illuminated the role of sociodemographic factors in shaping the observed behaviors. A significant negative association between education and BMI suggests that individuals with lower educational attainment may have faced greater challenges in maintaining healthy routines during the pandemic. A small but significant positive correlation between education and smoking status may reflect differential stress responses or occupational strain among more educated individuals. Moreover, the positive correlation between age and BMI and the negative association between age and honey consumption are consistent with well-established age-related behavioral patterns. These findings underscore the importance of integrating sociodemographic context into public health planning, as tailored interventions may be more effective in mitigating the long-term impact of pandemic-related behavioral changes.

The changes observed in oral hygiene and dietary behaviors may be partially explained by the unique conditions imposed during the COVID-19 lockdown. Restricted mobility, closure of non-essential shops, and fear of contagion in public spaces may have limited access to dental hygiene products such as dental floss or mouthwash [37,38]. Additionally, the interruption of routine dental appointments removed an important motivational and educational opportunity for reinforcing good oral care practices [39,40].

Also, some studies suggest that 6.8% of individuals have stopped worrying about their smile and oral health due to mask usage [41], and 32.49% discontinued their toothbrush routine [42]. In terms of diet, changes in daily structure, increased psychological stress, and a tendency towards comfort eating during confinement could explain the increased consumption of certain sugary products [43]. Financial constraints or reduced availability of specific food items may have also contributed to the variation in dietary habits. These factors together illustrate how the lockdown created an environment where both access and behavioral motivations were altered, thereby influencing self-care routines [38].

### Strengths and Limitations

When evaluating our study, it is essential to acknowledge both its strengths and its limitations. One notable limitation is the observational nature of the study design, which does not allow for the establishment of causal relationships. Furthermore, the data were collected through a self-administered questionnaire completed by the patients themselves. This means that the responses may not fully reflect the clinical reality, as they rely on the accuracy and honest self-reporting of the participants.

Another limitation relates to the assessment of oral hygiene habits. Although toothbrushing frequency was collected, the questionnaire did not include questions about the specific timing of brushing (e.g., after meals or food intake). This choice followed the structure recommended by the WHO’s Oral Health Surveys: Basic Methods (5th Edition), which focuses on brushing frequency rather than timing. However, we acknowledge that such information would be valuable in better understanding oral hygiene routines and recommend its inclusion in future studies exploring behavioral patterns more comprehensively. Nevertheless, the large sample size represents a major strength, enhancing the reliability and generalizability of the findings. Additionally, it is important to consider that changes in dental procedures due to the disruption of clinical activity during the COVID-19 pandemic posed further constraints to this study.

Despite these limitations, this study was conducted in accordance with internationally recognized guiding principles [17,44,45], which support its scientific value and credibility.

## 5. Conclusions

Considering the results and the characteristics of the studied population, we reject the null hypothesis of this study. Our findings suggest that the restrictions imposed during the COVID-19 pandemic had an unequal and multifaceted impact on oral health and dietary habits. Statistically significant changes were observed in health-related behaviors, including increased tobacco use and higher intake of coffee with sugar.

Although the overall mean score for tooth brushing frequency increased on the ordinal scale, the proportion of individuals brushing 2–3 times per day decreased, indicating a redistribution across frequency categories. Additionally, reductions in dental floss and mouthwash use were observed.

These changes were not uniformly distributed across the population: sociodemographic factors such as age, education, and gender were associated with greater vulnerability to negative behavioral shifts.

These findings underscore the importance of developing targeted public health strategies that address behavioral disparities and access barriers, particularly during periods of lifestyle disruption such as a pandemic.

## Figures and Tables

**Table 1:** Sociodemographic, health, and behavior characterization of the participants (*n* = 3380).

Variable	Total (*n* = 3380)	Pre-Lockdown (*n* = 2278)	Post-Lockdown (*n* = 1102)
Sex, % (*n*)			
Female	59.4 (2007)	60.1 (1369)	56.8 (476)
Male	40.6 (1373)	39.9 (909)	43.2 (362)
Age, mean	43.21	43.36	42.90
Age interval (years), % (*n*)			
18–24	21.3 (719)	21.2 (484)	21.3 (235)
25–44	32.4 (1094)	32.3 (736)	32.5 (358)
45–64	32.2 (1088)	31.9 (727)	32.8 (361)
≥65	14.2 (479)	14.5 (331)	13.4 (148)
Education, % (*n*)			
No studies	22.4 (756)	24.1 (550)	18.7 (206)
Elementary	37.4 (1265)	37.7 (859)	36.8 (406)
Middle	26.9 (1247)	37.4 (853)	35.8 (394)
Higher	3.3 (112)	0.7 (16)	8.7 (96)
BMI (Kg/m^2^) % (*n*)			
<18.5	3.6 (120)	3.8 (86)	3.1 (34)
18.5–24.9	46.1 (1558)	44.5 (1013)	49.5 (545)
25.0–29.9	34.1 (1153)	34.4 (784)	33.5 (369)
≥30.0	16.2 (549)	17.3 (35)	14.0 (154)
Active Smoker % (*n*)			
No	79.2 (2678)	82.0 (1869)	73.4 (809)
Yes	20.8 (702)	18.0 (409)	26.6 (293)

Abbreviations: BMI—Body Mass Index; *n*—number of participants.

**Table 2:** Descriptive data on oral hygiene practices (*n* = 3380).

Variable	Total (*n* = 3380)	Pre-Lockdown (*n* = 2278)	Post-Lockdown (*n* = 1102)
Type of toothbrush, % (*n*)			
Manual			
Yes	88.6 (2993)	88.5 (2017)	88.6 (976)
No	11.4 (387)	11.5 (261)	11.4 (126)
Electric			
Yes	10.9 (368)	10.8 (246)	11.1 (122)
No	89.1 (3012)	89.2 (2032)	88.9 (980)
Toothbrush frequency % (*n*)			
2–3 times/daily	75.6 (2555)	81.2 (1847)	64.2 (708)
1 time/daily	15.0 (507)	15.5 (352)	14.1 (155)
2–6 times/weekly	7.3 (248)	1.9 (44)	18.5 (204)
Never	2.1 (70)	1.5 (35)	3.2 (35)
Dental Floss usage, % (*n*)			
No	65.8 (2225)	62.0 (1413)	73.7 (812)
Yes	34.2 (1155)	38.0 (865)	26.3 (290)
Mouth wash usage, % (*n*)			
No	56.9 (1924)	55.5 (1264)	59.9 (660)
Yes	43.1 (1456)	44.5 (1014)	40.1 (442)

**Table 3:** Descriptive data on dietary habits consumption (*n* = 3380).

Variable	Total (*n* = 3380)	Pre-Lockdown (*n* = 2278)	Post-Lockdown (*n* = 1102)
Fresh fruit % (*n*)			
Several times a day	44.7 (1512)	43.8 (997)	46.7 (515)
Every day	30.5 (1031)	30.4 (693)	30.7 (338)
Once a week	18.3 (618)	19.1 (435)	16.6 (183)
Several times a month	2.7 (90)	2.7 (61)	2.6 (29)
Seldom/never	3.8 (129)	4.0 (92)	3.4 (37)
Biscuits and cakes % (*n*)			
Not know/not answer	0.1 (3)	0 (0)	0.3 (3)
Several times a day	6.3 (213)	5.9 (134)	7.2 (79)
Every day	17.8 (600)	18.8 (428)	15.6 (172)
Once a week	44.1 (1490)	44.2 (1007)	43.8 (483)
Several times a month	4.0 (135)	4.3 (97)	3.4 (38)
Seldom/never	27.8 (939)	26.9 (612)	29.7 (327)
Jam or Honey % (*n*)			
Not know/not answer	0.1 (3)	0 (0)	0.3 (3)
Several times a day	1.1 (37)	1.2 (28)	0.8 (9)
Every day	7.7 (260)	7.0 (160)	9.1 (100)
Once a week	19.9 (673)	19.4 (441)	21.1 (232)
Several times a month	2.0 (67)	2.0 (46)	1.9 (21)
Seldom/never	69.2 (2340)	70.4 (1603)	66.9 (737)
Sweets/candies, % (*n*)			
Not know/not answer	0.1 (2)	0 (0)	0.2 (2)
Several times a day	5.6 (190)	5.4 (122)	6.2 (68)
Every day	12.9 (436)	13.2 (300)	12.3 (136)
Once a week	44.1 (1490)	43.4 (988)	45.6 (502)
Several times a month	3.6 (123)	3.5 (79)	4.0 (44)
Seldom/never	33.7 (1139)	34.6 (789)	31.8 (350)
Lemonade or soft drinks, % (*n*)			
Not know/not answer	0.1 (3)	0 (0)	0.3 (3)
Several times a day	6.4 (216)	6.2 (141)	6.8 (75)
Every day	9.7 (327)	10.2 (233)	8.5 (94)
Once a week	26.0 (878)	25.9 (591)	26.0 (287)
Several times a month	3.3 (111)	3.3 (75)	3.3 (36)
Seldom/never	54.6 (1845)	54.3 (1238)	55.1 (607)
Tea with sugar, % (*n*)			
Not know/not answer	0.2 (6)	0 (0)	0.35 (6)
Several times a day	2.8 (95)	2.8 (64)	2.8 (31)
Every day	7.0 (237)	7.1 (161)	6.9 (76)
Once a week	10.4 (352)	10.7 (244)	9.8 (108)
Several times a month	1.5 (51)	1.2 (27)	2.2 (24)
Seldom/never	78.1 (2639)	78.2 (1782)	77.8 (857)
Coffee with sugar, % (*n*)			
Not know/not answer	5.3 (178)	0 (0)	16.2 (178)
Several times a day	21.0 (711)	22.5 (512)	18.1 (199)
Every day	15.2 (513)	16.3 (372)	12.8 (141)
Once a week	5.3 (180)	6.0 (136)	4.0 (44)
Several times a month	1.3 (43)	1.3 (29)	1.3 (14)
Seldom/never	51.9 (1755)	54.0 (1229)	47.7 (526)

**Table 4:** Results of independent samples *t*-tests comparing oral health and dietary habits before (*n* = 2278) and after lockdown (*n* = 1102). Total *n* = 3380.

Variable	Mean (Pre-Lockdown)	Mean (Post-Lockdown)	*p*-Value
BMI	1.65	1.58	0.007 *
Active smoker	0.18	0.27	<0.001 *
Fresh fruit consumption	2.32	2.24	0.076
Biscuits	3.90	4.01	0.152
Honey/Jam	5.19	5.06	0.013 *
Candy chewing	5.39	5.46	0.199
Sweets	4.23	4.17	0.199
Soft drinks	4.69	4.73	0.247
Tea with sugar	5.31	5.29	0.772
Coffee with sugar	4.15	3.58	<0.001 *
Manual toothbrush	0.89	0.89	0.984
Electric toothbrush	0.11	0.11	0.406
Dental floss usage	0.38	0.26	<0.001 *
Mouthwash usage	0.45	0.40	0.015 *
Tooth brushing frequency	0.24	0.62	<0.001 *

Categorical and ordinal variables were transformed into numerical scores for statistical purposes. Binary variables (e.g., smoking status, dental floss usage) were coded as 0 = No and 1 = Yes. Ordinal variables (e.g., dietary frequency, tooth brushing frequency) were assigned ascending numerical values based on frequency. Statistically significant differences (*p* < 0.05) are marked with an asterisk.

**Table 5:** Statistically significant Spearman correlations between sociodemographic and behavioral variables.

Variable	Spearman’s ρ	*p*-Value
Education—BMI	−0.22	<0.001
Education—Smoking status	+0.04	0.023
Age—Honey consumption	−0.12	<0.001
Gender—Smoking status	−0.11	<0.001
Age—BMI	+0.36	<0.001

Spearman’s rank correlation coefficient (ρ) was used to assess associations. Negative values indicate inverse relationships. All *p*-values < 0.05 were considered statistically significant.

## Data Availability

The entirety of the data compiled and examined throughout this study can be found within this document. Any additional queries should be directed towards the author in charge of correspondence.
